# Abducens Nerve in Patients with Type 3 Duane’s Retraction Syndrome

**DOI:** 10.1371/journal.pone.0150670

**Published:** 2016-06-28

**Authors:** Hee Kyung Yang, Jae Hyoung Kim, Jeong-Min Hwang

**Affiliations:** 1 Department of Ophthalmology, Seoul National University College of Medicine, Seoul National University Bundang Hospital, Seongnam, Korea; 2 Department of Radiology, Seoul National University College of Medicine, Seoul National University Bundang Hospital, Seongnam, Korea; NIH/NEI, UNITED STATES

## Abstract

**Background:**

We have previously reported that the presence of the abducens nerve was variable in patients with type 3 Duane’s retraction syndrome (DRS), being present in 2 of 5 eyes (40%) and absent in 3 (60%) on magnetic resonance imaging (MRI). The previous study included only 5 eyes with unilateral DRS type 3.

**Objectives:**

To supplement existing scarce pathologic information by evaluating the presence of the abducens nerve using high resolution thin-section MRI system in a larger number of patients with DRS type 3, thus to provide further insight into the pathogenesis of DRS.

**Data Extraction:**

A retrospective review of medical records on ophthalmologic examination and high resolution thin-section MRI at the brainstem level and orbit was performed. A total of 31 patients who showed the typical signs of DRS type 3, including abduction and adduction deficit, globe retraction, narrowing of fissure on adduction and upshoot and/or downshoot, were included. The abducens nerve and any other extraocular muscle abnormalities discovered by MRI were noted.

**Results:**

DRS was unilateral in 26 patients (84%) and bilateral in 5 patients (16%). Two out of 5 bilateral patients had DRS type 3 in the right eye and DRS type 1 in the left eye. Of the 34 affected orbits with DRS type 3 in 31 patients, the abducens nerve was absent or hypoplastic in 31 eyes (91%) and present in 3 eyes (9%). Patients with a present abducens nerve showed more limitation in adduction compared to patients with an absent abducens nerve (P = 0.030).

**Conclusions:**

The abducens nerve is absent or hypoplastic in 91% of DRS type 3. Patients with a present abducens nerve showed more prominent limitation of adduction. As DRS type 3 partly share the same pathophysiology with type 1 and 2 DRS, the classification of DRS may have to be revised according to MRI findings.

## Introduction

Duane’s retraction syndrome (DRS) consists of a congenital limited abduction and/or adduction, and narrowing of the palpebral fissure, eyeball retraction, and upshoots or downshoots of the affected eye on adduction.[1] DRS type 1 is characterized by limited abduction, whereas type 2, limited adduction, and type 3, limited abduction and adduction.[2] In 2005, Kim and Hwang evaluated the structure of the cranial nerves in 23 patients with DRS and found that the ipsilateral abducens nerve was absent in all patients with DRS type 1 and present in all patients with DRS type 2.[3] However, in patients with DRS type 3, the presence of the abducens nerve was variable, being present in 2 of 5 eyes (40%) and absent in 3 (60%).[3] The previous study included only 5 eyes with unilateral DRS type 3.[3] In addition, there has been only one pathologic report of DRS type 3 showing an absent abducens nerve in one case.[4] The purpose of this study was to supplement existing scarce pathologic information by evaluating the presence of the abducens nerve using high resolution thin-section MRI system in a larger number of patients with DRS type 3, thus to provide further insight into the pathogenesis of DRS.

## Patients and Methods

A retrospective review of medical records was performed on patients with the typical signs of DRS type 3 who visited the Department of Ophthalmology, Seoul National University Bundang Hospital between the years 2004 to 2014. Among 40 patients with the diagnosis of DRS type 3, 31 patients had performed MRI. Ophthalmologic examination and MRI findings at the brainstem level and the orbit were noted.[2] Diagnostic criteria of DRS type 3 included limited abduction and adduction, narrowing of fissure on adduction, globe retraction, and upshoot and/or downshoot. The five patients included in the previous report [3] were also included in this study. Patients were excluded if they did not have abnormal motility since birth and if the adduction or abduction deficit was related to other causative diseases, such as infantile esotropia or exotropia, third nerve palsy, sixth nerve palsy, internuclear ophthalmoplegia, synergistic divergence, or Möbius syndrome.

Ophthalmologic examinations of ductions and versions together with alternate prism cover tests at 6 cardinal gazes were performed by JMH or HKY. The evaluation of MRI test results was performed by JHK, who was blinded to the type of DRS of each patient. MRI was conducted using a 1.5 tesla system (Gyroscan Intera; Philips, Healthcare, Best, the Netherlands) in 8 patients and a 3 tesla system (Achieva; Philips, Healthcare, Best, the Netherlands) in 23 patients, with a SENSE (sensitivity encoding) head coil. Thin-section T2-weighted imaging was performed to visualize the cisternal segment of the abducens nerve in an axial plane through the brainstem, including the upper medulla oblongata and the pons. A 3-dimensional balanced turbo field echo sequence was used as previously described.[5] The presence or absence of the abducens nerve was evaluated according to previous studies.[3,5] If the entire cisternal segment of the nerve was identified, we considered it to be present. Secondly, if patients had unilateral DRS type 3 based on clinical records and the nerve was present in the affected side, we compared its size visually with the contralateral normal nerve. If it was identified in a definitely smaller size, it was considered to be hypoplastic. The right and left extraocular muscles were compared based on a side-by-side visual evaluation of their size and shape on the coronal T2-weighted images.

Statistical analyses were performed using SPSS for Windows (Ver. 22.0, Statistical Package for the Social Sciences, SPSS Inc., Chicago, IL). As the variables did not show a normal distribution, nonparametric methods including the Mann-Whitney U test and Fisher’s exact test were used to compare characteristics among groups. *P* values < .05 were considered statistically significant. This study adhered to the Declaration of Helsinki and the protocol was approved by the Institutional Review Board of Seoul National University Bundang Hospital. All clinical investigation was conducted according to the principles expressed in the Declaration of Helsinki. Informed consent was not given, as patient records and information were anonymized and de-identified prior to analysis.

## Results

Of the 31 patients with DRS type 3, the mean age was 20.4±19.6 years (range, 1–73). There were 15 males (48%) and 16 females (52%). DRS was unilateral in 26 patients (84%) and bilateral in 5 patients (16%). Two out of the 5 bilateral patients had DRS type 3 in the right eye and DRS type 1 in the left eye, and the other 3 patients had bilateral DRS type 3. Of the 26 unilateral patients, the right eye was affected in 8 patients (31%), and the left eye, in 16 patients (69%). Exotropia was found in 19 patients (61%) and esotropia in 3 patients (10%). Hypertropia of the affected eye was found in 5 patients (16%) and hypotropia in 4 patients (13%).

All of the extraocular muscles, including the lateral rectus muscles, appeared normal in size and symmetrical in shape in all patients on coronal T2-weighted images. Of the unilaterally affected 26 patients, the abducens nerve on the affected side was absent (n = 22) or hypoplastic (n = 1) in 23 patients (88%) and present in 3 patients (12%). The abducens nerve was absent in all 5 patients with bilateral DRS. Therefore, of the 34 affected orbits with DRS type 3, the abducens nerve was absent or hypoplastic in 31 eyes (91%) and present in 3 eyes (9%).

The clinical manifestations of patients with DRS type 3 were compared between patients with a present abducens nerve and an absent or hypoplastic abducens nerve (Table 1). Patients with a present abducens nerve showed more limitation in adduction compared to patients with an absent or hypoplastic nerve (*P* = 0.030, Mann-Whitney U test). The type of horizontal strabismus, vertical strabismus and the degree of abduction limitation were not significantly different between groups. Dissociated vertical deviation was found in only 1 patient with an absent abducens nerve.

**Table 1:** Clinical manifestations of patients with type 3 Duane retraction syndrome were compared between patients with a present abducens nerve (present group) and absent/hypoplastic abducens nerve (absent or hypoplastic group).

		Present group (n = 3)	Absent or Hypoplastic group (n = 28)	*P* value
Age at presentation(y)		9.7±2.1	21.6±20.3	0.710^a^
Male gender		1 (33%)	14 (50%)	0.525^b^
Bilateral		0 (0%)	5 (18%)	>0.999^b^
Unilateral		3 (100%)	23 (82%)	>0.999^b^
Laterality	Right eye	1 (33%)	7 (25%)	>0.999^b^
	Left eye	2 (67%)	16 (57%)	
Horizontal strabismus		1 (33%)	21 (75%)	0.195^b^
Type of horizontal strabismus	Exotropia	1 (33%)	18 (64%)	>0.999^b^
	Esotropia	0 (0%)	3 (11%)	
Vertical strabismus		2 (67%)	7 (25%)	0.195^b^
Type of vertical strabismus	Hypertropia^d^	1 (33%)	4 (14%)	0.293^b^
	Hypotropia^d^	1 (33%)	3 (11%)	
**Adduction limitation**^c^		3.7±0.6	2.1±1.1	**0.030**^a^
Abduction limitation^c^		2.7±1.5	3.2±0.7	0.681^a^
DVD		0 (0%)	1 (3.6%)	0.903^b^

y = years; Dissociated Vertical Deviation = DVD, Significant factors are expressed in bold characters

^a^ *P* value by Mann-Whitney U test

^b^ *P* value by Fisher’s exact test

^c^ Ocular motility assessment for adduction and abduction limitation was graded based on a subjective scale (0–4) of underaction

^d^ Vertical strabismus of the affected eye.

## Discussion

This study is, to our knowledge, the largest series of DRS type 3, including the former 5 patients reported in our previous study[3]. Unlike the previous study [3] which showed that 40% had a present abducens nerve in DRS type 3, most of the patients in this study showed the same MR finding of DRS type 1, the absent abducens nerve. Since the first MR report of the absent abducens nerve by Parsa et al,[6] there have been four studies that evaluated the presence of the abducens nerve according to the type of DRS. Among them, the studies of Xia et al’s [7] and Denis et al’s [8,9] included only type 1 and 2, and not DRS type 3. Therefore, there have been only two studies covering all the three types of DRS. We have previously reported that the abducens nerve on the affected side was present in all of DRS type 2, absent in all of DRS type 1, and either present or absent in DRS type 3.[3] This was the only study to show a sensitivity of 100% in normal controls for detecting the abducens nerve by MRI.[3] Yonghong et al[10] reported an absent or hypoplastic abducens nerve in all 5 patients with DRS type 1, 1 patient with DRS type 2, and 4 patients with DRS type 3. In addition, there have been two case series of DRS type 3. [11, 12] She et al [11] showed an absent or hypoplastic abducens nerve in all 5 patients with DRS type 3. Demer et al [12] also reported absent or hypoplastic intraorbital and intracranial abducens nerves in 3 out of 7 eyes with Duane-radial ray syndrome showing limited abduction and adduction. Combining the data of all previously reported cases of DRS type 3, the likelihood to observe an intact abducens nerve on MRI would be 7 out of 50 eyes (14%). In other words, if MR imaging in a patient with both adduction and abduction deficit shows an intact abducens nerves, the likelihood of having DRS type 3 would obviously be low, but not zero.

As in the previous reports of DRS, the abducens nerve could be small or hypoplastic and not completely absent. However, in our study, the abducens nerve was completely absent in all but one patient in the absent group, who had a relatively hypoplastic nerve compared to the normal contralateral side. If the patient is affected unilaterally, discrimination between a normal and hypoplastic nerve can easily be done by the comparison with the normal contralateral side. In bilateral DRS cases, both sides of the abducens nerve may be affected and in these cases, a cutoff value to distinguish a normal and hypoplastic nerve by the absolute thickness or the ratio of the affected and unaffected side may be helpful, which remains to be elucidated. In our study, all 5 cases of bilateral DRS consisted of type 3 and/or type 1, and the abducens nerve was absent on both sides. We cannot exclude the possibility that a very hypoplastic nerve smaller than the section size (0.25–0.5 mm) might exist in these cases. However, our study protocol with a 3 Tesla system and a section thickness of 0.25–0.5 mm is far beyond the practical guidelines of brain MRI, and we may carefully interpret this as an absent nerve until higher spatial resolutions with higher field MRI could clarify this issue. The inconsistent thickness of the nerve along the entire cisternal segment and variable age ranges may also confuse interpretation of the thickness of the abducens nerve.

Regarding the mechanism of DRS type 3, there is a pathologic report of one case which showed an absent abducens nerve on the affected side and a hypoplastic contralateral abducens nerve.[13] Using electromyography, Huber [2] showed intense synchronous discharges of both medial and lateral rectus muscles on adduction and abduction in patients with DRS type 3. Different types of anomalous lateral rectus muscle innervations in DRS type 3 have been recorded since Huber, and even DRS type 1 appeared to have various innervation patterns. [14] The amount of fibers that abandon the medial rectus nerve for supply to the lateral rectus is variable, resulting in variable clinical manifestations. In this concept, DRS types 1 and 3 may be considered as a continuum. As the number of fibers that abandon the medial rectus nerve increases, which leads this muscle to lose force and the lateral rectus to gain force in its abnormal contraction, until arriving to the situation in which their forces equalize themselves (symmetric co-contraction).[14] In this situation, there is no adduction or abduction, the bridle effect of the co-contraction is maximal and consequently the retraction and the anomalous vertical movements are more evident, as in DRS type 3. As the absence of the abducens nerve is common in both DRS type 1 and the absent group of DRS type 3, this implies that the clinical distinction between DRS type 1 and type 3 may be artificial and that the two subtypes of DRS actually share the same pathophysiology. Conversely, the increase of the amount of residual innervation of the abducens nerve leads to DRS type 2, and the abduction may become near to normal. The more prominent adduction limitation and less abduction limitation (although not statistically significant) together with a present abducens nerve found in the present group of DRS type 3 patients also suggests its common pathophysiology with DRS type 2. Vertical misalignment is also more often observed in patients with DRS type 2,[14] which is consistent with our results that patients with a present abducens nerve showed more vertical strabismus in the primary position (67% vs. 25%) although this did not reach statistical significance due to the small number of patients. Unfortunately, these clinical characteristics overlap in a relatively wide range between groups. The grading of abduction/adduction limitations largely depends on the examiner’s subjective judgment and it may also change over time. Although the limited resolution of MRI precludes confirmation of actual innervation patterns at the level of extraocular muscle fibers, the intimate contact of a branch of the oculomotor nerve with the lateral rectus may suggest its dysinnervation to the lateral rectus.[12,15] Therefore, the classification of DRS can be enhanced with imaging, but there is not enough data here to explain how MRI could be used to change the classification.

Differential diagnosis of DRS from other congenital abduction deficits is not complicated when patients show the characteristic features of DRS, such as narrowing of the palpebral fissure, eyeball retraction and upshoots or downshoots. However, some children with DRS present only with an abduction deficit,[16] and adults with DRS who present with sudden diplopia in adulthood could be misdiagnosed as acquired sixth nerve palsy.[17] A diagnosis of DRS also could be challenging in a patient with atypical strange eye movements [18–20] or if the patient insists that the abduction deficit developed after trauma.[21] Therefore, an absent intracranial abducens nerve would strongly suggest DRS type 1 or 3, which could be useful for the differential diagnosis of an abduction deficit.

Regarding DVD, it is not common for patients with DRS to show DVD.[22, 23] DVD is usually observed in patients who have experienced disruption of fusion in their early life and we assume that patients with DRS can develop and maintain fusion with abnormal head postures, mostly with face turn, therefore do not commonly develop DVD. Additionally, patients with DRS usually have tight rectus muscles which may act as a tether to elevation. The only patient with DVD in our report was the one who had DRS type 3 in his right eye and a large angle of constant exotropia since birth. He alternately fixed with both eyes and the DVD was more notable in the normal contralateral eye.

In conclusion, the abducens nerve is absent in 91% of the patients with DRS type 3. DRS type 3 patients with a present abducens nerve showed more limitation in adduction compared to the absent group. MR imaging verifying absent or hypoplastic abducens nerve strongly suggests DRS type 1 or 3, which could be useful for the differential diagnosis of an abduction deficit in complex extraocular movement disorders. From the results of our study, DRS type 3 may partly share the same pathophysiology with DRS type 1 and type 2, and the classification of DRS may have to be revised according to MRI findings.
